# DamID profiling of dynamic Polycomb-binding sites in *Drosophila* imaginal disc development and tumorigenesis

**DOI:** 10.1186/s13072-018-0196-y

**Published:** 2018-06-05

**Authors:** Marco La Fortezza, Giovanna Grigolon, Andrea Cosolo, Alexey Pinduyrin, Laura Breimann, Helmut Blum, Bas van Steensel, Anne-Kathrin Classen

**Affiliations:** 1 Faculty of Biology, Ludwig-Maximilians-University Munich, Grosshaderner Strasse 2-4, 82152 Planegg, Martinsried, Germany; 2 Department of Environmental Systems Science, ETH Zurich, Universitätstrasse 16, 8092 Zurich, Switzerland; 3 Department of Health Sciences and Technology, ETH Zurich, Schorenstrasse 16, 8603 Schwerzenbach, Switzerland; 4 Center for Biological Systems Analysis, Albert-Ludwigs-University Freiburg, Habsburgerstrasse 49, 79104 Freiburg, Germany; 5 Institute of Molecular and Cellular Biology, Siberian Branch of Russian Academy of Sciences, Acad. Lavrentiev Ave. 8/2, Novosibirsk, 630090 Russia; 6 Max-Delbrück-Center for Molecular Medicine (MDC), Robert-Rössle-Str. 10, 13092 Berlin, Germany; 7 Laboratory for Functional Genome Analysis, Gene Center Munich, Ludwig-Maximilians-University Munich, Feodor-Lynen-Str. 25, 81377 Munich, Germany; 8 Division Gene Regulation, The Netherlands Cancer Institute, Plesmanlaan 121, 1066 CX Amsterdam, The Netherlands

**Keywords:** DamID, Wing imaginal disc, Polycomb, *Scrib*

## Abstract

**Background:**

Tracking dynamic protein–chromatin interactions in vivo is key to unravel transcriptional and epigenetic transitions in development and disease. However, limited availability and heterogeneous tissue composition of in vivo source material impose challenges on many experimental approaches.

**Results:**

Here we adapt cell-type-specific DamID-seq profiling for use in *Drosophila* imaginal discs and make FLP/FRT-based induction accessible to GAL driver-mediated targeting of specific cell lineages. In a proof-of-principle approach, we utilize ubiquitous DamID expression to describe dynamic transitions of Polycomb-binding sites during wing imaginal disc development and in a *scrib* tumorigenesis model. We identify *Atf3* and *Ets21C* as novel Polycomb target genes involved in *scrib* tumorigenesis and suggest that target gene regulation by Atf3 and AP-1 transcription factors, as well as modulation of insulator function, plays crucial roles in dynamic Polycomb-binding at target sites. We establish these findings by DamID-seq analysis of wing imaginal disc samples derived from 10 larvae.

**Conclusions:**

Our study opens avenues for robust profiling of small cell population in imaginal discs in vivo and provides insights into epigenetic changes underlying transcriptional responses to tumorigenic transformation.

**Electronic supplementary material:**

The online version of this article (10.1186/s13072-018-0196-y) contains supplementary material, which is available to authorized users.

## Background

Understanding the in vivo dynamics of DNA binding by chromatin regulatory proteins is key to elucidate the molecular basis of cell behaviours ranging from differentiation to adaptation and plasticity. The model system *Drosophila* has contributed tremendously to our understanding of chromatin dynamics during developmental transitions, stem cell differentiation and also tumorigenesis. Yet, like other in vivo model systems, the small size and the heterogeneous fate composition of *Drosophila* tissues still pose challenges to the detailed tracking of DNA binding sites in different cell populations and lineages in vivo.

Several experimental approaches to overcome these challenges have been developed. For example, chromatin immunoprecipitation (ChIP) protocols use fluorescence-activated cell sorting (FACS) or immunoprecipitation (IP)-based methods to isolate *Drosophila* cell populations from tissues [1–4]. These approaches, however, still require a significant amount of input material for reproducible results, which has prevented these methods from being used in contexts where small source tissues, such as imaginal discs, are routinely isolated by manual dissection. Alternatively, recent publications establish cell-type-specific DamID profiling in *Drosophila* brains [5–8]. PCR-amplified tracking of adenine methylation (m6A) conferred by DamID to GATC sequence motifs and the absence of IP steps significantly reduces the input material required for DamID [9]. Moreover, m6A is only generated in cell types expressing DamID constructs; therefore, DamID protocols do not necessitate to physically isolate cell populations from complex tissues [5, 6]. Thus, DamID is a very attractive technology to profile small and even rare cell populations in vivo.

We wanted to adapt the inducible FRT/FLP-out DamID system described for *Drosophila* brains [5] to cell-type-specific profiling in imaginal discs. These small tissues have a rich history as model to study developmental patterning, tumorigenesis and regeneration [10] but are mostly accessed by manual dissection for experimental analysis. We wanted to establish versatility of targeting DamID expression to specific cell types by enabling the use of GAL4 driver lines available in these tissues. While the TaDa-DamID system [6, 8] also utilizes cell-type-specific targeting by GAL4 drivers, TaDa depends on acute expression patterns of a chosen GAL4 driver at the time of analysis. In contrast, we aimed to target DamID to specific cell lineages enabling tracking of DNA binding sites in parental and descendant populations—independent of whether the GAL4 driver used was still active in descendant cells. Furthermore, while the FRT/FLP-out DamID has been suggested to be compatible with GAL4-dependent targeting [7], its cell-type specificity and experimental feasibility have not yet been tested. Finally, we sought to establish a proof of principle that a limiting amount of manually dissected imaginal disc material is sufficient to sensitively detect changes in DNA binding activity in development and disease.

More specifically, we asked whether DamID may be suitable to track the epigenetic regulator Polycomb (Pc) in wing imaginal discs (WIDs) during different developmental stages and tumorigenic transformation. Polycomb is the founding member of the Polycomb group (PcG) family of proteins who form different complexes, such as the Polycomb Repressive Complexes 1 and 2 (PRC1 and PRC2). PcG proteins epigenetically silence genes required for fate specification, cell cycle progression and tissue growth by modulating multiple histone modifications [11–15]. Previous studies demonstrated that PcG protein binding sites change dynamically throughout early embryonic development and suggested that a number of Pc target genes, like JAK/STAT cytokines of the *unpaired (upd)* family, may be silenced by Pc to suppress tumorigenesis [16–20]. In fact, a significant overlap between PcG target genes and genes upregulated in neoplastic WIDs mutant for the epithelial polarity regulator *scribbled (scrib)* has been described [17]. However, direct experimental evidence for dynamic Pc-binding at co-regulated candidate genes is still outstanding.

We report here the co-regulation of multiple oncogenic genes by dynamic Pc-binding, while also identifying at least two novel Pc target genes involved in *scrib* tumorigenesis. We furthermore suggest that gene regulation by Atf3 and AP1 transcription factors as well as modulation of insulator function plays crucial roles in dynamic PcG behaviour. We establish these findings by DamID-seq analysis of wing imaginal discs samples derived from as little as 10 larvae. We furthermore describe a versatile GAL4-driven cell lineage-specific DamID system that can be used for DamID-seq profiling in many *Drosophila* tissue.

## Results

### Establishment of versatile GAL4-dependent control of cell lineage-specific DamID

**Fig. 1:**
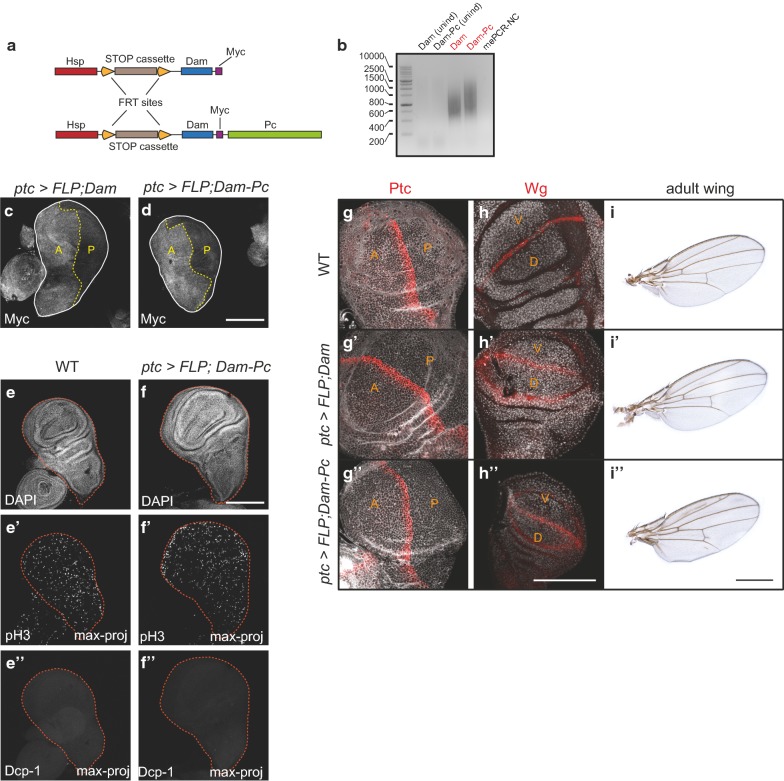
Establishing cell lineage-specific DamID in wing imaginal discs. **a** Schematic representation of the FLP-inducible Dam and Dam-Pc constructs used in this study. **b** Characteristic DNA smear formed by DamID methylation-dependent PCR products on agarose gel. Lanes 1–2: wing imaginal disc (WID) samples, where FLP expression has not been induced. Lanes 3–4: WID samples from genotypes ubiquitously expressing FLP after induction by a heat shock (*hsflp*). me-PCR-NC refers to negative PCR controls lacking DNA template. **c**, **d** WIDs stained for expression of the Myc-tag if Dam (**c**) and Dam-Pc (**d**) were induced by *ptcGAL4*-driven expression *of UAS*-*FLP(EXEL)*. Expression of the Myc-tagged fusion proteins was boosted by a heat shock (see Experimental procedures). A and P refer to anterior and posterior compartments, respectively. **e**–**e″** Wild-type WID stained with DAPI (**e**), and for pH3 (**e′**) and Dcp-1 (**e″**). Maximum projections of a confocal stack are shown in E’ and E’’ to reveal all signals. **f**–**f″** WID from *ptc *> *FLP;Dam*-*Pc* expressing larvae stained with DAPI (F) and for pH3 (**f**′) and Dcp-1 (**f″**). Maximum projections of a confocal stack are shown in **f′** and **f″** to reveal all signals. **g**–**g″** WIDs from indicated genotypes stained for patched (Ptc). A and P refer to anterior and posterior compartments, respectively. **h–h″** WIDs from indicated genotypes stained for wingless (Wg). D and V refer to dorsal and ventral compartments, respectively. **i**–**i″** Adult wings from indicated genotypes 24 h after eclosion. All scale bars: 100 µm

To establish DamID in WIDs, we employed a transgenic fly line carrying an inducible Dam or Dam-Pc fusion construct [5, 7]. Briefly, a full-length *Hsp70* promoter is separated from the *Dam* or the *Dam*-*Pc* coding sequence by a cassette containing a transcriptional terminator flanked by FRT sites, which prevents transcription of Dam or Dam fusion proteins (Fig. 1a). Ubiquitous or cell-type-specific expression of a FLIP recombinase (FLP) mediates site-directed recombination of flanking FRT sites and removal of the terminator cassette, allowing expression of Dam or Dam fusion proteins [5, 7]. Indeed, only upon ubiquitous expression of a heat-shock-induced FLP, we observed the characteristic DNA smear formed by the methylation-dependent PCR products amplified from genomic DNA (gDNA) extracted from WIDs (Fig. 1b, Additional file 1: Fig. S1A). In addition, genotyping PCR confirmed the genomic elimination of the terminator cassette from the DamID constructs only after FLP induction (Additional file 1: Fig. S1B, B′). Combined, these observations indicate that the terminator cassette prevents transcription of Dam or Dam-Pc proteins in WIDs and that their expression can be efficiently induced by the presence of FLP.

We wanted to optimize this inducible DamID system for flexible cell-type-specific targeting by the rich repertoire of GAL4 driver lines available. We thus screened a number of *UAS*-*FLP* constructs from different sources for their ability to mediate efficient removal of the FRT-flanked transcriptional terminator cassette. Moreover, we specifically searched for a *UAS*-*FLP* line that did not show leaky expression in the absence of a GAL4 driver to prevent unspecific removal of the terminator cassette. Indeed, combining a *UAS*-*FLP(JD2)* transgene [21] with the inducible DamID system caused GAL4-independed removal of the terminator cassette (Additional file 1: Fig. S1B′). In contrast, a *UAS*-*FLP(EXEL)* transgene [22] did not induce removal of the terminator cassette in WIDs in the absence of a GAL4 driver (Additional file 1: Fig. S1B″). Only combining a *DamID;UAS*-*FLP(EXEL*) line with a *rotund(rn)GAL4 driver* caused partial removal of the terminator cassette in WIDs, consistent with the restricted expression of *rnGAL4* in the central domain of the disc (Additional file 1: Fig. S1B″). This region was visualized using the G-trace system (Additional file 1: Fig. S1C) [23], which maps cell lineage history and real-time expression of a GAL4 driver of choice. To prove that Dam and Dam-Pc fusion proteins are really expressed in a cell-type-specific and GAL4/UAS-FLP(EXEL)-dependent manner, we sought to visualize expression of the Myc-tag encoded by both constructs [5, 7]. To this end, we induced removal of the terminator cassette by crossing a stable *DamID;UAS*-*FLP(EXEL)* line to a *patched*(*ptc)* GAL4 driver. *ptcGAL4* is active in a row of cells anterior to the anterior–posterior compartment boundary in WIDs (Additional file 1: Fig. S1C′). However, most of the anterior compartment derives from cells that had expressed *ptc* earlier during development (Additional file 1: Fig. S1C′). Thus, the early removal of the terminator cassette during development under the control of *ptcGAL4* is expected to cause expression of Myc-tagged Dam and Dam-Pc proteins in all cells of the anterior WID compartment. Notably, Dam and Dam-Pc proteins expressed under the control of the heat-shock promoter are present at undetectable levels if flies were kept at 21 °C. However, if boosted by a heat shock (see Experimental procedures), high expression of the Myc-tag could be detected specifically in the anterior compartment, if FLP expression was induced by *ptcGAL4* (Fig. 1c, d). Importantly, Myc-tag expression was completely absent in the posterior compartment. Similarly, when DamID was induced using the posterior compartment driver *engrailed*(*en)*GAL4, boosted expression of the Myc-tag was exclusively detected in the posterior compartment (data not shown). These results indicate that *UAS*-*FLP(EXEL)* allows for the specific and flexible induction of cell-type-specific DamID in WIDs under the versatile control of cell-type-specific GAL4 drivers.

High expression levels of Dam are known to interfere with DamID specificity [24] and viability [5] (Additional file 1: Fig.S1D, E). Therefore, to understand whether expression of Dam by the low basal activity of the *Hsp70* promoter at 21 °C is suitable for DamID profiling by maintaining wing disc cell viability, we monitored the occurrence of mitosis and apoptosis by immunodetection of phospho-H3S10 (pH3) and the activated effector caspase Dcp-1, respectively. No differences in mitotic or apoptotic activity between the anterior and posterior compartment could be observed when larvae were maintained at 21 °C and the terminator cassette was removed under the control of *ptcGAL4/UAS*-*FLP(EXEL)* (Fig. 1e–f″). Furthermore, immunodetection of developmental regulators such as Ptc itself (Fig. 1g–g″) or wingless (Wg) (Fig. 1h–h″) revealed appropriate patterning activity, and adult wings arising from these discs displayed only subtle alterations, such as extra vein tissues (Fig. 1i–i″). Combined these results suggest that inducible DamID profiling does not interfere with WID viability and developmental progression and thus presents an excellent option for cell-type-specific mapping of DNA binding sites in WIDs in vivo.

### DamID and ChIP profiles of Polycomb-binding sites correlate

**Fig. 2:**
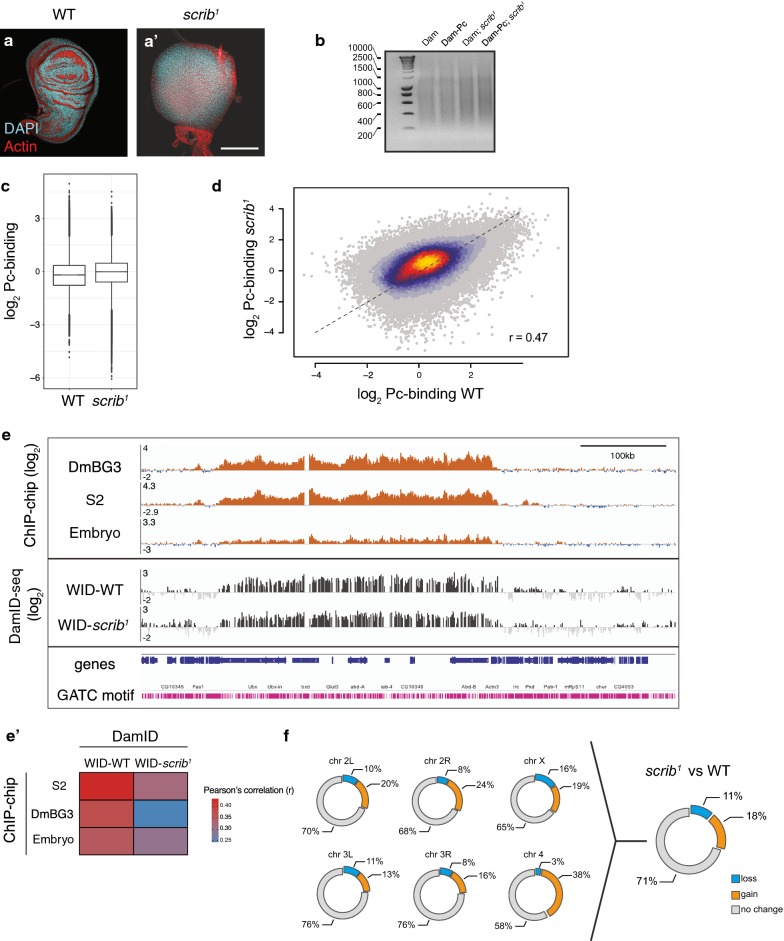
DamID and ChIP profiles of Polycomb-binding sites correlate. **a**–**a′** Wild-type WID (**a**) and *scrib*^*1*^ WID stained with DAPI (cyan) and phalloidin (red). Scale bar: 100 µm. **b** Characteristic DNA smear formed by DamID methylation-dependent PCR products obtained from *hsflp*-induced samples isolated from WT WIDs (lane 1 and 2) or *scrib*^*1*^ WIDs (lanes 3 and 4). **c** Box plot comparing the distribution of the Pc-binding intensities (log_2_) at individual GATC fragments (normalized to Dam) in WT and *scrib*^*1*^ DamID-seq samples. Pc-binding intensities averaged over two biological replicates are shown. **d** Heat-scatterplot showing the correlation of Pc-binding intensities (log_2_) at individual GATC fragments (normalized to Dam) in WT and *scrib*^*1*^. (Pearson’s correlation, r = 0.47). **e** ChIP-chip Pc-binding profiles (modENCODE) from three different sources (S2 cells, DmBG3 cells and embryo) and DamID-seq profiles mapped to individual GATC fragments obtained in this study (WT and *scrib*^*1*^ WID) visualized across the *BX*-*C* cluster (demarcated by dotted lines). GATC motifs mapping to the genome sequence are indicated below. **e′** Pearson’s correlations for a comparison of Pc-binding intensities in ChIP-chip profiles (modENCODE) from three different sources (S2 cells, DmBG3 cells and embryo) and Pc-binding intensities WT and *scrib*^*1*^ WID DamID-seq Pc profiles at GATC fragments mapping to microarray probe sequences. **f** Percentage of genomic sites in *scrib*^*1*^ compared to WT WID that lose (*loss*), acquired new (*gain*) and had no change (*no change*) in Pc-binding visualized for each chromosome and the whole genome. *Loss*, *gain* and *no*-*change* transitions were determined by transitions between ‘*enriched*’, ‘*intermediate’* and ‘*depleted’* Pc-binding states classified by a three-state HMM analysis. Note that the *no*-*change* category contains GATC fragments that were classified as ‘*enriched*’, ‘*intermediate’* and ‘*depleted’* for Pc-binding and thus includes Pc target and non-target genes

To provide a proof of principle that DamID sensitively detects differences in DNA binding activity in vivo, we wanted to compare Pc-binding profiles between wild-type (WT) and *scrib*^*1*^ tumourous wing discs (Fig. 2a, a′, Additional file 1: Fig. S2). We used *scrib*^*1*^ as a classic example of a polarity-deficient tumour suppressor gene [25] for which genetic interactions with and defects in Polycomb silencing have been reported [17].

We first induced ubiquitous expression of Dam and Dam-Pc in whole larvae using a FLP under the control of a heat-shock promoter (*hsflp*). We isolated and amplified methylated genomic DNA from WIDs of 10 WT or *scrib*^*1*^ third-instar larvae expressing either Dam alone or a Dam-Pc fusion protein (Fig. 2b) and generated NGS libraries using protocols devoid of additional PCR amplification steps to avoid PCR biases (see Experimental procedures).

The PCR-free NGS library preparation from 20 WIDs generated sequencing profiles with relatively low correlation coefficients across replicates (Additional file 1: Fig. S1F), likely due to high noise in profiles. However, assessment of multiple reproducibility parameters, such as correlation coefficients (Additional file 1: Fig. S1F), hierarchical clustering approaches using 94 DamID-Seq profiles (Additional file 1: Fig. S3A) and autocorrelation of neighbouring GATC sites at Lag 2 (Additional file 1: Fig. S3B) [26], revealed that technical replicates within genotypes are always more similar to each other than replicates across genotypes. Thus, PCR-free DamID-seq libraries can reproducibly reveal DNA binding profiles for small in vivo tissue samples.

While a subset of PcG target genes was previously reported to be upregulated in *scrib*^*1*^ WIDs [17], we found that total levels of H3K27 modifications were comparable between WT and *scrib*^*1*^ WIDs (Additional file 1: Fig. S4A). Our DamID-seq profiles confirmed that Pc-binding at individual sites (as defined by any genomic sequences flanked by Dam-targeted GATC motifs, also referred to as GATC fragments hereafter) was not globally altered in *scrib*^*1*^ (Fig. 2c). Indeed, when the genome-wide distribution of Pc-binding intensities at these sites was compared, the correlation between WT and *scrib*^*1*^ discs (Pearson’s correlation,* r* = 0.47, Fig. 2d) was only slightly lower than for biological replicates (Pearson’s correlation *r* = 0.51, Additional file 1: Fig.S1F). Importantly, broad binding of Pc to the *Bithorax* complex *(BX*-*C)* observed in Pc-DamID profiles could also be detected in Pc ChIP profiles from S2 cells, DmBG3 cells and whole embryo (Fig. 2e) [27, 28]. The Pearson’s correlation coefficients calculated for a comparison of the genome-wide Pc-binding intensities at individual GATC fragments in our Pc-DamID-seq and the corresponding GATC fragments in individual Pc ChIP-chip profiles ranged from 0.25 to 0.4 (Fig. 2e′). This finding is in agreement with previous comparisons of the two techniques [29–31] (for example Pearson’s correlation *r* = 0.37 in [30]). Our analysis thus indicates that DamID-seq is a suitable method to reveal DNA binding profiles of Polycomb in WID in vivo.

### Polycomb-binding is altered only at a subset of target sites in *scrib*^*1*^ wing discs

To understand whether alterations in Pc-binding at specific target genes may contribute to tumour phenotypes in *scrib*^*1*^ disc, we performed a three-state hidden Markov model (HMM) analysis of Pc-binding at individual GATC fragments to define *‘depleted’, ‘intermediate’* and *‘enriched’* Pc-binding states and analysed transitions between these states when comparing *scrib*^*1*^ to WT WIDs (see Experimental procedures, Additional file 1: Fig. S4B, Additional files 2: SF2 and 3: SF3) [26, 32–35]. As expected, we obtained three possible clusters that described the changes between the two profiles, namely (1) ‘*no change’,* which defined GATC fragments that did not vary in their Pc-binding classification between WT and *scrib*^*1*^ profiles, irrespective of whether these sites were bound by Pc in WT and *scrib*^*1*^ WIDs or not; (2) *‘loss’* defined GATC fragments, which were bound by Pc in WT but not in *scrib*^*1*^ discs; and (3) ‘*gain’* defined GATC fragments, which were not bound by Pc in WT but in *scrib*^*1*^ WID samples. This analysis revealed that about 11% of *‘intermediate’* and *‘enriched’* Pc-binding states present in WT were lost in *scrib*^*1*^ WIDs and about 18% of *scrib*^*1*^ *‘intermediate’* and *‘enriched’* Pc-binding states were arising de novo (Fig. 2f). This suggests that Pc-binding dynamics are altered in a loci-specific manner in *scrib*^*1*^ discs.

**Fig. 3:**
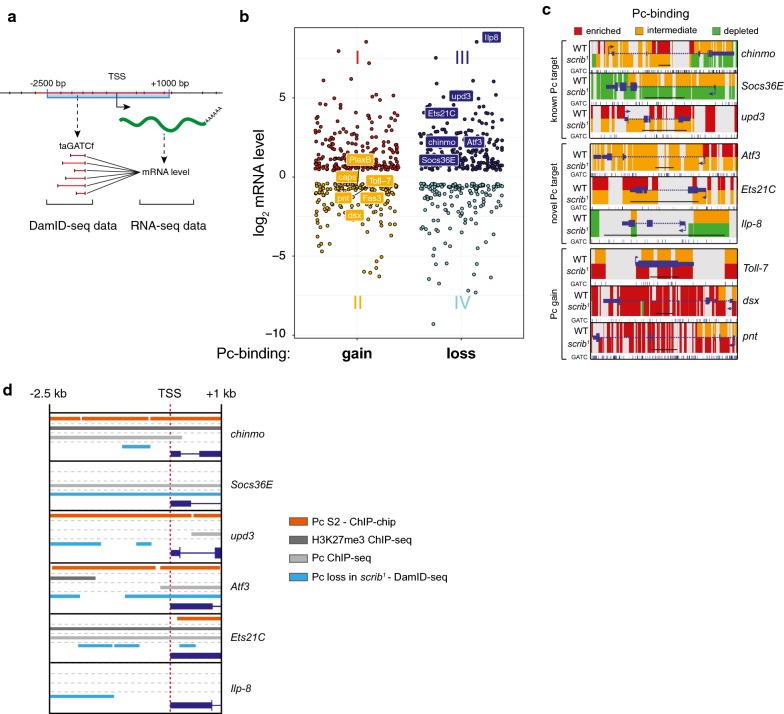
Polycomb-binding is altered only at specific *loci* in *scrib*^*1*^ wing discs. **a** Schematic representation of the workflow used to analyse transition in Pc-binding states on transcription-associated GATC fragments (taGATCf) that are mapping to a regulatory region surrounding a TSSs of a gene that was differentially expressed in *scrib*^*1*^ versus WT RNA-seq samples. **b** Graph visualizes the distribution of GATC fragments classified according to a *gain* or *loss* in Pc-binding in *sc*–*rib*^*1*^ compared to WT profiles, and according to the change in expression level of the gene in *sc*–*rib*^*1*^ to whose TSS the GATC fragment had been mapped to. Group I (RNA—upregulated; Pc-binding—gain); group II (RNA—downregulated; Pc-binding—gain); group III (RNA—upregulated; Pc-binding—loss); group IV (RNA—downregulated; Pc-binding—loss). **c** Profiles visualize Pc-binding in WT and *scrib*^*1*^ WIDs at indicated loci that represent known Pc target genes involved in tumorigenesis, and novel Pc target genes belonging to group II and III loci. Pc-binding levels on each GATC fragments were classified by a three-state HMM analysis to be either ‘*enriched*’ (red), ‘*intermediate’* (orange) and ‘*depleted’* (green) and visualized by centring a fragment around individual GATC motifs. GATC fragments not recovered by our DamID-Seq analysis in either genotype are shown in grey and were excluded for both genotypes in our analysis. Intron–exon structure, TSS and position of GATC motifs are indicated for each gene. Scalebar is 5 kb. **d** Profiles visualize the presumptive regulatory region 2.5 kb upstream to 1.5 kb downstream of the TSS of indicated genes. Domains bound by Pc in S2 cells (modENCODE) (orange), domains enriched for H3K27me3 (dark grey) and Pc (light grey) by ChIP-Seq analysis in wing discs [42] and domains with *loss* transitions DamID_Seq profiles in *scrib*^*1*^ (light blue)

To learn more about the effects that gain and loss of Pc-binding may have on transcriptional activity of Pc target genes in *scrib*^*1*^ discs, we related DamID Pc-binding sites to previously published WT and *scrib*^*1*^ WID transcriptome dataset [17]. To this end, we extracted the presumptive regulatory region spanning across the transcriptional start site (TSS)(− 2.5 kb ~ + 1 kb) of all genes differentially expressed in *scrib*^*1 *^(Fig. 3a) and recovered all included GATC fragments, hereafter referred to as transcription-associated GATC fragments (taGATCf) (Additional file 1: Fig. S4C). We compared changes in Pc-binding (*gain, loss* or *no change*) at an individual taGATCf with changes in the transcription levels of the associated differentially expressed gene (Fig. 3a). When comparing WT and *scrib*^*1*^ WIDs, many transcriptional changes at differentially expressed genes whose presumptive regulatory region contained at least one Pc-bound taGATCf occurred in the absence of changes to Pc-binding (data not shown). In numerous instances, however, a gain or loss of Pc-binding at any one taGATCf was linked to a gain or loss in transcript levels of the associated gene (Fig. 3b, Additional file 1: Fig. S4D, Additional file 4: Table S1). Surprisingly, we found that, in some cases, gain in Pc-binding could occur in the context of upregulated transcription (group I) and loss of Pc-binding could occur when transcription was downregulated (group IV) (Fig. 3b). While this unexpected behaviour appears to contradict the established role of Pc as promoter of gene silencing, we speculate that, instead, additional regulatory inputs at these target sites dominate target gene expression or, alternatively, that the bulk of transcriptional changes and changes in Pc-binding states may arise in two different cell populations.

### Polycomb-binding at differentiation and tumour-associated targets is altered in *scrib*^*1*^ discs

Our approach indicated the presence of multiple genes associated with transcriptional upregulation upon loss of Pc-binding (group III) and with transcriptional repression upon gain of Pc-binding (group II) in *scrib*^*1 *^(Fig. 3b), which is consistent with the described function of Pc in gene silencing [11–15]. We thus focused our subsequent analysis on these genes.

Surprisingly, group II included genes implicated in axon guidance, for example *dsx, Lrt, caps, PlexB, pdm3, Toll*-*7* and *Fas3 *(Fig. 3b), possibly reflecting a failure to develop wing and thorax sensory neurons. While all group II genes gained Pc-binding for at least one taGATCf in *scrib*^*1*^ discs, we wanted to provide additional evidence for a role of PcG in regulating their expression. An analysis of transcript levels in WIDs mutant for the PRC1 components *Psc/Su(z)2* [17] revealed that specifically *dsx*, *Toll*-*7* and the neuronal Notch target *pnt* were upregulated upon loss of repressive PcG complex function (Fig. 3c, Additional file 1: Fig. S4E). This suggests that at least a subset of group II genes are *bona fide* Pc target genes.

**Fig. 4:**
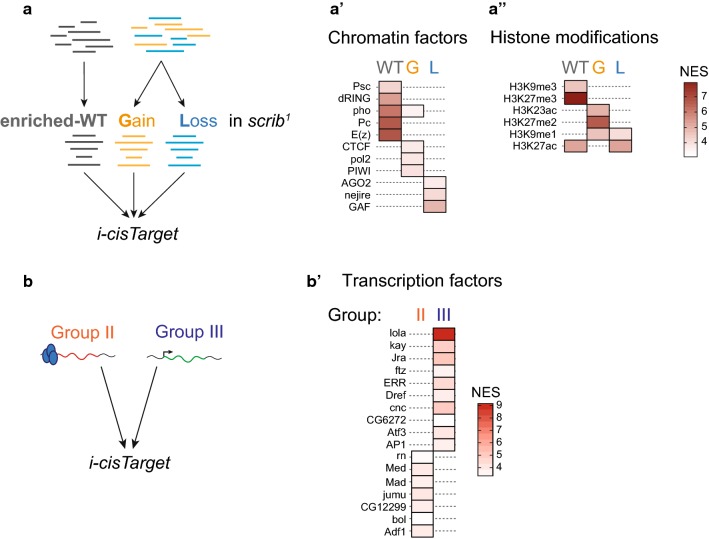
Modulation of Polycomb-binding and target gene expression is associated with enrichment of specific regulatory elements. **a** Schematic representation of the workflow used to identify regulatory elements in GATC fragments pooled into categories representing *no change* (nc), *gain* (G) or *loss* (L) of *‘enriched’* Pc-binding states in *scrib*^*1*^ DamID-seq profiles if compared to WT. Note that the ‘*no*-*change’* category for this conservative analysis only contains GATC fragments that were classified as ‘*enriched*’ for Pc-binding and thus excludes the *‘depleted’* and ‘*intermediate’* classifications. **a′**–**a″)** Regulatory elements identified by *i*-*cisTarget* that either represent enrichment for chromatin-binding factors (**a′)** or presence of specific histone modifications (**a″**) at GATC fragments *‘enriched’* for Pc-binding (**a**) that show *no change* (nc), *gain* (G) or *loss* (L) of Pc-binding in *scrib*^*1*^ WIDs. Normalized enrichment scores (NES) are visualized as coloured scale. **b** Schematic workflow used to identify enriched regulatory elements within genomic regions spanning 2.5 kb upstream to 1.5 kb downstream of the TSS in group II and III genes (see Fig. 3b for definition). **b′**–**b″)** Enrichment for transcription factors identified by *i*-*cisTarget* in the presumptive regulatory domains of Pc-targeted genes belonging to group II or III

Strikingly, however, group III was comprised of many genes implicated in promoting tumorigenic transformation, but which had not yet been identified as Pc target genes. Foremost among them are *Ets21C* [36, 37], *Atf3* [38] and *Ilp8* [39, 40]. As reported previously, we also found the tumour-associated genes *upd3* [16, 17], *SOCS36E* [16, 41, 42] and *chinmo* [43, 44] to be Pc target genes (Fig. 3b, c). *Ets21C*, *Atf3*, *Ilp8* and *upd3* are known JNK target genes [37, 45], whereas *chinmo* and *SOCS36E* are important effectors of JAK/STAT signalling [41, 43]. Importantly, Pc-binding at all but one gene can also be identified in Pc ChIP profiles from S2 cells (Fig. 3d). A recent study [42] suggests that a large number of PRC1 targets involved in proliferation and signalling, like *SOCS36E*, may only acquire PRC1-binding but not PRC2-dependent H3K27me3 modifications. We thus specifically asked whether H3K27me3 and Pc may be found at *Ets21C*, *Atf3* and *Ilp8* loci in WT WIDs. To do so, we compared our data with H3K27me3 and Pc ChIP-seq profiles published by Loubiere et al. [42] (Fig. 4b). Like *chinmo* [42], *Ets21C* and *Atf3* carry both H3K27me3 and Pc signatures (Fig. 3d), suggesting that *Ets21C* and *Atf3* may be canonical PcG target genes utilizing PRC2-dependent H3K27me3 modifications for transcriptional regulation. On the other hand, like *SOCS36E* [42], *upd3* only acquires PRC1-binding but lacks H3K27me3 (Fig. 3d). Interestingly, neither H3K27me3 nor Pc signatures from previous studies mapped to *Ilp8* (Fig. 3d).

Despite these different behaviours with respect to H3K27me3 modifications, *Ets21C*, *Atf3, Ilp8, SOCS36E, upd3* and *chinmo* are all upregulated upon loss of repressive PRC1 complex function in *Psc/Su(z)2* mutant WIDs, demonstrating a role for Pc in silencing these tissue-stress-responsive genes in wild-type WIDs (Additional file 1: Fig. S3D). Thus, we identify at least three tumour-associated genes as novel *bona fide* Pc target genes and imply that the tumour-suppressive function of PcG proteins [16] integrates with regulation by the two important tumour-promoting pathways JNK and JAK/STAT.

### Modulation of Polycomb-binding and target gene expression is associated with enrichment of specific regulatory elements

A question we wanted to address is how epigenetic mechanisms may intersect with changes in signalling environment of cells, and more specifically, how Pc-binding may be affected by cross-talk with transcription factors that act as effectors of signalling cascades activated during tumorigenesis. Thus, to advance our insight into how gain or loss of Pc-binding in *scrib*^*1*^ WIDs may be regulated, we analysed GATC fragments classified by the three-state HMM analysis to be *‘enriched’* in Pc-binding, for predicted transcription factor binding motifs or modENCODE-identified chromatin domains [27, 46] using *i*-*cisTarget* [47] (see Experimental procedures). In parallel, we performed an *i*-*cisTarget* on GATC fragments classified as *gain* or *loss* of ‘enriched’ Pc-binding states in *scrib*^*1*^ WIDs (Fig. 4a). As expected, Pc-bound GATC fragments in WT were enriched for PRC1 and PRC2 binding, as well as H3K27me3 and H3K9me3 modifications (Fig. 4a′, a″). In contrast, regulatory regions exhibiting dynamic Pc-binding transitions in *scrib*^*1*^ displayed high NES scores for RNA-mediated silencing machineries (Piwi, Ago2), transcriptional activation by histone acetylation (Nejire/CBP) or recruitment of RNAPol II (Fig. 4a′), all of which may cooperate with CTCF (Fig. 4a′) in insulator-dependent transcriptional regulation and spatial organization of chromatin [48–52]. Interestingly, histone modifications previously observed to occur at genes that are expressed, but importantly, at intermediate levels [53], were also detected at dynamic Pc-binding sites (Fig. 4a″). This suggests that Pc target genes, which experience altered Pc-binding in *scrib*^*1*^, may be subject to transcriptional modulation rather than absolute repression by Pc.

Next, we wondered whether tumour-associated transcripts upregulated upon loss of Pc-binding in *scrib*^*1*^ (group III, Fig. 3b) were characterized by a specific signature of regulatory elements. We thus repeated an *i*-*cisTarget* analysis for the presumptive regulatory region spanning the transcriptional start site (TSS)(− 2.5 kb ~ + 1 kb) of genes belonging to group III (Fig. 4b). Strikingly, AP-1 (Jra/Kay), Atf3, Cnc and Lola-binding motifs enriched in group III loci (Fig. 4b′, Additional file 5: Table S2) and align with the stress-dependent activation of *chinmo, Atf3, Ets21C, Ilp8, upd3* and *SOCS36E* associated with high JNK and JAK/STAT activity during wound healing, regeneration and tumorigenesis [38, 44, 54–57].

We repeated an *i*-*cisTarget* analysis for group II genes, whose transcripts were downregulated upon gain of Pc-binding in *scrib*^*1*^ (Fig. 4b) to ask how Polycomb may be recruited to these sites. In agreement with the observation that group II genes were enriched for axon guidance targets, we found that transcription factors specifically expressed in neurons, such as Jumu and CG12299, were enriched in regulatory regions of group II (Fig. 4b′, Additional file 5: Table S2). Importantly, however, wing patterning regulators, such as the transcription factor Rn and the Dpp/TGF-β signalling effectors Med and Mad, were also enriched, confirming that wing differentiation is affected in a Polycomb-dependent manner in *scrib*^*1*^ WID (Fig. 4b′) [17]. These data, however, may indicate that transcriptional downregulation of genetic circuits involved in neuronal and wing disc patterning promotes binding of Pc to these target genes.

Based on our finding that GATC fragments gaining Pc-binding in *scrib*^*1*^ were enriched for CTCF (Fig. 4a′), we asked whether insulator elements locate to group II genes. Strikingly, 71% of group II genes contained Flybase-mapped class I and II insulator elements within their gene body. In contrast, insulator features mapped to only 19% of group III genes. This suggests that insulator-dependent modulation of Pc function or Pc-dependent modulation of insulator function may have important consequences for Pc-targeted gene expression in *scrib*^*1*^.

### Polycomb-binding transitions fail in *scrib*^*1*^ imaginal discs development

**Fig. 5:**
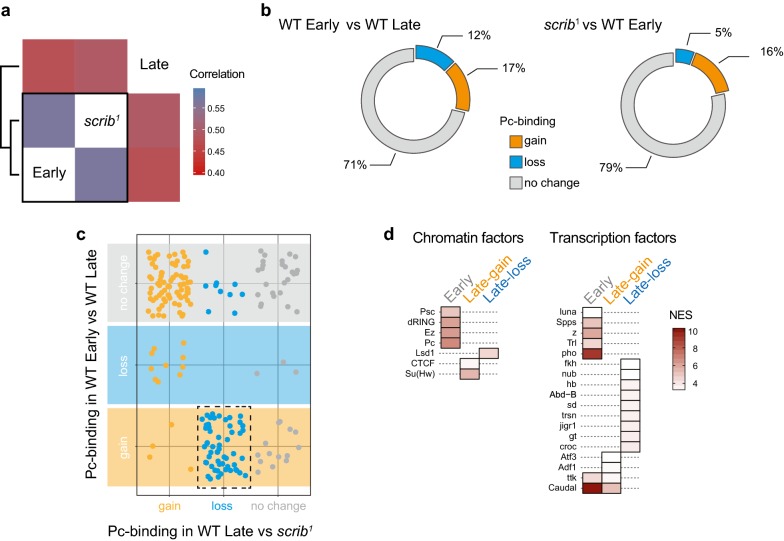
Polycomb-binding transitions fail in *scrib*^*1*^ imaginal discs development. **a** Pearson’s correlations between DamID-seq Pc profiles obtained from WIDs in early larval stages (Early), late larval stages (Late) and in *scrib*^*1*^. **b**–**b′** Percentage of GATC fragments that classify as *loss, gain* and *no change* in Pc-binding states in (**b**) late (WT Late) if compared to early (WT Early) WIDs and (**b′**) in *scrib*^*1*^ WIDs if compared to early (WT Early) WIDs. *Loss*, *gain* and *no*-*change* transitions were determined by transitions between ‘*enriched*’, ‘*intermediate’* and ‘*depleted’* Pc-binding states classified by a three-state HMM analysis. Note that the *no*-*change* category contains GATC fragments that were classified as ‘*enriched*’, ‘*intermediate’* and ‘*depleted’* for Pc-binding and thus includes Pc target and non-target genes. **c** Relationship of Pc-targeted GATC fragments that classify as *loss, gain* and *no change* in Pc-binding in ‘*scrib*^*1*^ if compared to late (WT Late) WIDs’ versus ‘late (WT Late) if compared to early (WT Early) WIDs’ (*gain*—orange*, loss*—light blue*, no change*—grey). The dotted frame highlights sites that lost Pc-binding in *scrib*^*1*^ if compared to WT Late samples but should have gained Pc-binding during normal wing disc development. **d** Regulatory elements identified by *i*-*cisTarget* that represent enrichment for chromatin-binding factors and transcription factors at GATC fragments conservatively classified as ‘*enriched*’ for Pc-binding in early WID samples and on GATC fragments classified as a *gain* (Late gain) or *loss* (Late loss) of Pc-binding by transitioning in and out of the ‘*enriched*’ state in late developmental stages if compared to an earlier stage. Normalized enrichment scores (NES) are visualized as coloured scale

Previous studies indicate that abnormal differentiation in *scrib*^*1*^ discs may be linked to deregulation of Pc function [17]. To better characterize the differentiation state of *scrib*^*1*^ discs, we asked whether *scrib*^*1*^ Pc-DamID profiles correlated better with developmentally younger than with older WIDs, indicative of a failure to acquire PcG-regulated wing fates during development. We thus compared Pc-DamID profiles from WT and *scrib*^*1*^ late third-instar WIDs to Pc-DamID profiles from young WT WIDs isolated 2 days earlier in development (120 h AEL at 21 °C, early third instar) (Additional file 6: SF4). Strikingly, Pc-DamID profiles of *scrib*^*1*^ WIDs correlated more strongly with young WIDs than with older WIDs (Fig. 5a). Importantly, while the percentage of Pc-‘enriched’ GATC fragments gained in *scrib*^*1*^ and older WT WIDs stayed relatively constant if compared to young WIDs, the percentage of Pc-‘enriched’ GATC fragments that was lost was strongly reduced in *scrib*^*1*^ (Fig. 5b). Furthermore, target sites that normally gained Pc-binding during development failed to gain Pc-binding in *scrib*^*1*^ WIDs (Figs. 2f, 5c). Combined, this suggests that early Pc-bound sites stay bound as *scrib*^*1*^ discs progress through development and that sites which should gain Pc-binding in older *scrib*^*1*^ discs fail to do so. These results imply that a failure to execute Pc-dependent fate specification may contribute to the lack of wing disc differentiation in *scrib*^*1*^ discs.

A subsequent *i*-*cisTarget* analysis of young WID profiles revealed that Pc-‘enriched’ GATC fragments in young WIDs displayed PRC1 and PRC2-binding, confirming that they are canonical Pc target sites (Fig. 5d). GATC fragments that specifically lost ‘enriched’ Pc-binding in late development scored high for binding sites of the wing differentiation regulators nubbin (Nub) and scalloped (Sd) (Fig. 5d), reflecting the expansion of the central wing domain. GATC fragments that gained Pc-binding in late development were enriched in binding sites for Atf3 and Adf1 (Fig. 5d). Adf1 was recently identified to be critical for recruitment and tethering of Pc to target sites [58]. The enrichment of Atf3 motifs may suggest that Atf3 target genes are increasingly silenced as wing discs development progresses, which has indeed been observed for Atf3 expression [59]. This may also have important implications for the reduction in regenerative capacity previously attributed to Pc silencing of critical tissue-stress-responsive enhancers in late WIDs [60].

However, GATC fragments with dynamic Pc transitions during development were also enriched for CTCF and Su(Hw) insulator components, as well as for the histone demethylase Lsd1. Combined, these invoke earlier observations of insulator signatures at dynamic Pc-targeted sites (Fig. 4a′) and imply that Pc-binding dynamics at insulator elements, which are critical for organization of chromatin in the nucleus [48–52], are crucial to Pc function during differentiation. Intriguingly, a detailed analysis of our DamID profiles revealed that the Pc-bound GATC fragment sizes recovered from earlier developmental stages were larger than those recovered from late imaginal discs (Additional file 1: Fig. S5). Moreover, in *scrib*^*1*^ datasets, GATC fragment sizes occupied an intermediate distribution (Additional file 1: Fig. S5). The size range differences cannot be recapitulated by Dam profiles alone (data not shown). It may suggest that Pc-binding to genome regions characterized by different GATC motif frequencies is developmentally regulated and may reflect different distributions at promoters, introns or intergenic regions. However, it may also suggest a link between changes to Pc-binding and chromatin accessibility, where chromatin compaction during development may reduce the likelihood of distant GATC motifs to be methylated by Pc-Dam fusion proteins.

## Discussion

As a consequence of the limited availability and accessibility of sample material, in vivo ChIP protocols are technically challenging [61]. Here, we report that DamID sensitively and reproducibly detects Pc-binding differences in wing imaginal discs with input samples derived from just 10 larvae. We propose that the lower limit necessary for good quality DamID profiles of imaginal discs is even less. For example, we specifically omitted PCR amplifications during preparation of NGS libraries to avoid oversampling of PCR biases. Consequently, we eliminated an opportunity to amplify weak signals to detectable levels. Indeed, published DamID-seq protocols report PCR amplification of NGS libraries without adverse effects [5, 8].

By targeting an ectopic signature to specific cells, FRT/FLP-out DamID circumvents the challenges of in vivo ChIP approaches that require the researcher to purify cell-type-specific nuclei from complex tissues. For this purpose, previously described cell-type-specific DamID systems rely either on the real-time expression patterns of GAL4 drivers (TaDa) or on cell-type-specific promoters that directly drive the expression of a FLP to achieve cell-type specificity [5–8]. In contrast, we describe a cell lineage-specific DamID system by utilizing a specific UAS-FLP(EXEL) that can be combined with any GAL4 driver for maximum flexibility to permanently target DamID to different cell types and their descendants. Genetic strategies based on individual GAL4 drivers can be optimized and validated by G-trace analysis to reveal temporal and spatial patterns of the GAL4-targeted lineage. Combined, the approach reported here opens the opportunity to track transitions of DNA binding sites in parent and daughter cell populations of a cell lineage over time.

Here we demonstrate that DamID sensitively detects significant changes in Pc-binding between three different source samples. While Pc silencing is not globally altered in a *scrib*^*1*^ mutant background, the transcriptional changes that correlated with altered Pc-binding at specific loci allowed us to identify three novel Pc target genes (*Atf3*, *Ets21C, Ilp8*), which are implicated in tissues stress responses and tumour growth in many proliferating tissues [36–38, 43, 44]. We find that Atf3, AP-1 (Jra/Kay) and Lola-binding sites are enriched at these genes that are activated in *scrib*^*1*^ mutant discs, suggesting that these transcriptional regulators [38, 44, 54, 55] may oppose Pc silencing to activate a PcG target gene network in tissue repair and tumorigenic transformation. Curiously, transcript levels of core PcG components are downregulated by stress-induced JNK signalling [62] and two core PRC1 transcripts are mildly reduced in *scrib*^*1*^ WIDs [17]. This downregulation of PcG may sensitize Pc target genes, such as *Atf3*, *Ets21C, Ilp8, upd3, SOCS36E* and *chinmo,* for activation in stress-induced or tumorigenic contexts.

Our findings furthermore imply the high correlation between *scrib*^*1*^ and younger WID profiles indicates that a failure of *scrib*^*1*^ WID to undergo Pc-dependent fate differentiation contribute to *scrib*^*1*^ phenotypes. Our analysis furthermore implies that such developmental transitions mediated by Pc may be associated with insulator dynamics that could mediate global changes to accessibility of Pc-regulated chromatin domains. How insulator dynamics may regulate dynamic Pc-binding during development needs to be clarified in future studies. Similarly, while our analysis focused on Pc dynamics in different tissue states, a recent study highlights large scale remodelling of HP1-dependent chromatin and of silent ‘black’ chromatin states in developmental transitions of neuron, which are also likely to play a role in imaginal disc development and tumorigenesis [63].

## Experimental procedures

### Fly stocks

All stocks and experimental crosses were maintained on standard fly food at 18  °C or 25  °C unless otherwise specified. The following transgenes and fly lines were used in this study:

- *y,w;Hsp70P(FRT.STOP#1)DamMyc(ZH51C*-*3xP3*-*RFP);*
- *y,w;Hsp70P(FRT.STOP#1)DamMycPc(ZH51C*-*3xP3*-*RFP);*
- *UAS*-*RedStinger,UAS*-*FLP.Exel3,Ubi*-*p63E(FRT.STOP)Stinger* (G-trace);
- *ptcGAL4 and ptcGAL4, tubGAL80* ^*ts*^ */CyO;*
- *rnGAL4; and rn[GAL4*-*DeltaS], tubGAL80*^*ts*^*/TM6c*
- *en*-*GAL4, UAS*-*GFP; tub*-*GAL80*^*ts*^
- *scrib*^*1*^;
- *hsflp*^*122*^;
- *UAS*-*FLP(JD2)*;
- *UAS*-*FLP(EXEL)(3)*

### Organismal induction of DamID constructs

Development of embryos was synchronized by an 8-h egg collection on standard fly food at 21 °C. FLP expression, which was controlled by a heat-shock promoter (*hsflp*), was induced by a 1-h temperature shift to 37 °C in a water bath. To analyse DamID profiles of young WIDs, a heat shock was performed at 3 days after egg lay (AEL). To analyse DamID profiles of late WIDs, a heat shock was performed at 5 days AEL. To account for the developmental delay characteristic of *scrib*^*1*^ homozygous animals, *scrib*^*1*^ larvae were heat-shocked at 6 days AEL. Afterwards, larvae were kept at 21 °C to maintain a low basal activity of the *Hsp70* heat-shock promoter driving expression of *Dam* and *Dam*-*Pc* transcripts. Wing imaginal discs were dissected 48 h after induction of *hsflp*. Genomic excision of the STOP cassette from DamID constructs as a result of FLP activity was tested with regular PCR protocols on gDNA extracted from WIDs (see below) using the primers *hhsp*-*int* (actgcaactactgaaatctgc) and *Dam*-*r* (cgctattgatatcggcaagg).

### Tissue dissection and genomic DNA extraction

Ten *Drosophila* larvae were dissected in cold Shields and Sang M3 medium, and WIDs were collected in 1.5-ml tubes on ice. Discs were resuspended in a total volume of 400 µl lysis buffer (10 mM Tris–HCl pH 8.0; 10 mM EDTA pH 8.0; 100 mM NaCl; 0.5% SDS) with proteinase K (20 mg/ml, NEB) and incubated for 4 h at 55 °C. Phenol–chloroform purification and RNase A (QIAGEN) digestion were followed up by a standard ethanol precipitation to obtain pure DNA. Each sample was subsequently run on 1% agarose gel to confirm DNA integrity and to estimate DNA concentrations. DNA from control and experimental samples was isolated at the same time and processed in parallel.

### DamID sample processing, PCR and NGS library preparation

Isolation of genomic DNA (gDNA) from WIDs is described above. For each condition and stage, two independent biological samples were processed and analysed as described in [7] with minor changes. Briefly, after gDNA extraction, 600 µg of gDNA was digested with DpnI restriction enzyme (10 U, New England Biolabs) with CutSmart buffer (New England Biolabs) in a total volume of 10 µl at 37 °C for 6 h. DpnI digestion was terminated with heat inactivation at 80 °C for 20 min. Digested fragments were ligated to 12.5 pmol DamID adapters with T4 ligase (Roche) with T4 ligase buffer in a total volume of 20 µl for 16 h at 16 °C. Ligated gDNA fragments were subsequently digested with DpnII (10 U, New England Biolabs) in DpnII buffer (New England Biolabs) in a total volume of 50 µl for 1 h at 37 °C. Ten microlitres of DpnII digested products was amplified by PCR using MyTaq Red Mix (Bioline) with 50 µM Adr-PCR primers in a total volume of 50 µl. PCR program: 10 min at 68 °C; 1 min at 94 °C, 5 min at 65 °C, 15 min at 68 °C; 1 min at 94 °C, 1 min at 65 °C, 10 min at 68 °C—repeated 3X; 1 min at 94 °C, 1 min at 65 °C, 2 min at 68 °C—repeated (17X). Twelve microlitres of PCR products was run on 1.5% agarose gel to examine the expected DNA smear. Primers and adaptors sequences are described in [64]. PCR products were purified using QIAquick PCR purification kit (QIAGEN) according to manufactures protocol. Samples were eluted in 50 µl of nuclease-free water. After purification, DNA concentration was determined with Qubit Fluorometric Quantitation (ThermoFisher) and adjusted to 20 ng/µl for all samples prior to libraries preparation for NGS. One microgram of DNA was transferred to a microTUBE AFA Fiber Screw-Cap 6 × 16 mm (Covaris) and sheared to an average size of around 350 bp, using a Covaris M220 focused-ultrasonicator with the following settings: duty factor = 20%, peak incident power = 50 W, cycles per burst = 200, time = 55 s, temperature = 6 °C. Illumina TruSeq PCR-free LT library preparation kit (Illumina) was used to obtain DamID-seq library according to manufactures protocol. Next-generation sequencing was run on Illumina GenomeAnalyzer IIx cBot machine. fastq file analysis was performed according to methods described in [5].

### Bioinformatic tools—general information

Bioinformatic analysis was performed using *R* (v. 3.4.0) (https://www.r-project.org/) and *bedtools* (v. 2.26.0) software (http://bedtools.readthedocs.io/en/latest/#). Analysis for enriched regulatory elements was performed using *i*-*cisTarget* (https://gbiomed.kuleuven.be/apps/lcb/i-cisTarget/index.php) [47].

### Identification and characterization of Pc-bound target sites

DamID-seq fastq files were processed as described previously [5] with the following two modifications. The mapping of reads onto GATC fragments by the software ‘HTSeq-count’ was performed with a higher stringency criterion (by using the ‘intersection_strict’ instead of ‘union’ overlap resolution mode). GATC fragments showing highly discordant values between replicates were excluded from the analysis as described [65].

Pc-binding sites (‘bound’ targets) were identified based on Dam-normalized log2-transformed DamID-seq profiles by fitting a three-state hidden Markov model (HMM) to define ‘*enriched*’, ‘*intermediate’* and ‘*depleted’* Pc-binding states for each GATC fragment, as described previously (Additional files 2: SF2, 3: SF3, 6: SF4) [26, 32–35]. We chose a three-state model to avoid random assignment of *intermediate* binding to either ‘*enriched*’ or ‘*depleted’* states [26]. Thus, while ‘*intermediate’* states could arise for any biological, genetic or technical reasons, we could distinguish them in our analysis.

As the lengths of the genomic GATC fragments (bins) are not of equal size, we used the BioHMM algorithm, a heterogeneous HMM, which takes into account the distance between adjacent bins [66]. This algorithm was previously implemented in the Bioconductor package snapCGH [67, 68]. We adapted the BioHMM algorithm for identification of three Pc-binding states (‘*enriched*’, ‘*intermediate’* and ‘*depleted’*). The R code of adapted BioHMM algorithm is provided as Additional file 7: SF1. The three-state HMM analysis outputs of each GATC fragment were compared between ‘WT’ and ‘*scrib’* datasets, as well as between ‘early’ and ‘late’ development in WT, to assess the dynamics of Polycomb-binding between two samples. To maintain the directionality of differences, the result of this comparison was reported as either ‘*gain*’, ‘*loss*’ or ‘*no change*’ for each GATC fragment between ‘*enriched*’, ‘*intermediate’* and ‘*depleted’* HMM states.

### RNA-seq and ChIP-chip data analysis

RNA-seq datasets were obtained from [17]. Genes were selected for further analysis according to the statistical significance (adjusted *p* val<* 0.05*) and subsequently divided in *upregulated* and *downregulated* expression according to the change in transcript levels. Differential gene expression was provided as log_2_ of the fold change between *WT, scrib*^*1*^ and *Psc/Su(z)2*^*XL26*^ datasets. ChIP-chip datasets were downloaded from the modENCODE repository (http://www.modencode.org/): Pc in S2 cells (ID 3791), Pc in DmBG3 cells (ID 325), Pc in embryo (ID 3957). Sequence overlap of microarray probe sequences in ChIP-chip datasets and Dam-normalized GATC fragments in DamID-Seq datasets was analysed using *bedtool intersect* function. Pearson’s correlation between DamID-seq and ChIP-chip data was calculated by correlating the intensity of Dam-normalized Pc-binding at each GATC fragment in either WT or *scrib*^*1*^ datasets to the intensity of Pc-binding at the corresponding microarray probe for the respective Pc ChIP-chip analysis from S2 cells, DmBG3 cells or embryo.

### Transcription-associated GATC fragments (taGATCf)

Regulatory regions associated with genes differentially expressed in *scrib*^*1*^ were defined as genomic regions spanning 2.5 kb upstream to 1.5 kb downstream of the transcriptional start sites (TSS) of the selected genes. Briefly, the coordinates of the regulatory regions were calculated from the TSS coordinates and the strand on which the TSS mapped on. This information was acquired from Flybase (*Batch Download*, http://flybase.org) (genome annotation *dm6*) using the FB.ID of all differentially expressed genes. Subsequently, genome coordinates of GATC fragments were converted into the appropriate genome annotation (*dm3* → *dm6*, LiftOver tool—UCSC, https://genome.ucsc.edu/cgi-bin/hgLiftOver) and mapped to the regulatory regions using the *intersect* function in *bedtools2* (no limitations were considered on the amount of overlap between the two coordinates’ sets). Only GATC fragments that overlapped with selected regulatory regions were defined as transcription-associated GATC fragments (taGATCf) and used for the comparative analysis of DamID-seq and RNA-seq data in wild-type and *scrib*^*1*^ WIDs (Fig. 3a). Subsequently, regulatory regions mapping to *upregulated* or *downregulated* genes were further subdivided according to transitions in Pc-binding at each of their associated taGATCf (‘*gain*’, ‘*loss*’ or ‘*no change*’ for each GATC fragment between ‘*enriched*’, ‘*intermediate’* and ‘*depleted’* HMM states). The entire regulatory region was subsequently classified as *gain* in Pc-binding, if one or more taGATCf within this region ‘*gained’* Pc-binding and other taGATC fragments displayed *‘no change’*. Conversely, a regulatory region was classified as *loss* in Pc-binding, if one or more taGATCf within this region ‘*lost’* Pc-binding and other taGATC fragments displayed *‘no change’*. Finally, regulatory regions which contained a mix of taGATCf with both *gain* and *loss* HHM states were classified as *mixed (m1*-*low mRNA levels, m2*-*high mRNA levels*, Additional file 1: Fig. S3.D) and not considered in subsequent analysis.

As a result, the described method subdivides regulatory regions into the following four groups: *group I (RNA*—*up regulated; Pc-binding*—*gain); group II (RNA*—*down regulated; Pc-binding*—*gain); group III (RNA*—*up regulated; Pc-binding*—*loss); and group IV (RNA*—*down regulated; Pc-binding*—*loss).*

### Analysis for enriched regulatory elements using *i*-*cisTarget*

We performed our *i*-*cisTarget* analysis adhering to an enrichment score threshold = 2 and rank threshold = 10,000. We defined significantly enriched motifs by setting the normalized enrichment scores (NES) > 3. For factors with multiple enriched motifs, we selected only the one with the highest NES. The following features (Databases 3.0 of *i*-*cisTarget*) were selected during the analysis: PWMs, TF binding sites, non-TF binding sites, histone modifications. These parameters were common to all *icis*-Target analysis.

#### icis-Target analysis on GATC fragments with assigned HMM transitions:

Figure 4a–a″: this analysis was performed on pools of GATC fragments with the following defined HMM transition states: *gain* or *loss* of Pc-binding in *scrib*^*1*^ by transitioning in and out of ‘*enriched*’ HMM states, and *no change* in Pc-binding in *scrib*^*1*^ by staying ‘*enriched*’ (excluding *‘depleted’* and ‘*intermediate’* HMM states).

Figure 5d: one analysis was performed on GATC fragments that were defined as *‘enriched’* in WT Early profiles after the three-state HMM analysis (Fig. 5d). Another analysis was performed on pools of GATC fragments with *gain* or *loss* of Pc-binding in ‘WT Late’ discs by in and out of ‘*enriched*’ HMM states.

#### icis-Target analysis on taGATCf fragments mapping to the presumptive regulatory region of Pc-targeted genes:

Sequences of all regulatory regions established for the analysis of taGATCf were first converted to a genome annotation suitable for *icis*-Target analysis (*dm6* → *dm3*) and then subdivided into their respective group (group I, group II, group III and group IV). The *icis*-Target analysis was performed on groups II and III independently (Fig. 4c–c″).

### Immunohistochemistry

To detect the Myc-tagged Dam proteins, expression of *Dam* and *Dam*-*Pc* constructs was boosted by a heat shock for 1 h at 37 °C 6 h prior to dissection to strongly induce the *Hsp70* promoter. This heat shock induces abnormally high Dam and Dam-Pc expression levels that can be detected by immunohistochemistry but are unsuitable for genomic DamID profiling and reduce cell viability. Larvae were dissected and cuticles were fixed for 15 min at room temperature in 4% paraformaldehyde (PFA). Washing steps were performed in 0.1% Triton X-100/PBS (PBT). The following antibodies were incubated overnight at 4 °C: rabbit α-Dcp-1 (1:500, Cell Signalling), mouse α-H3S10p (1:2000, Abcam), mouse α-Myc (1:50, DSHB). Secondary antibodies (Molecular Probes), DAPI and phalloidin-TRITC (Sigma) were incubated at room temperature for 2 h. Experimental and control samples were processed together and imaged on the same microscope (Leica TCS SP-5).

### Adult wing imaging

Adult flies were collected 12 h after eclosion and stored in 2-propanol. Wings were dissected and mounted in Euparal (Sigma) on regular slides for microscopy. Imaging was done using a stereoscopic zoom microscope (Nikon, SMZ745).

## Additional files

**Additional file 1.** Supplemental figures S1–S5.

**Additional file 2: SF2.** WIG file containing the results of the three-state HMM analysis of wild type wing imaginal discs in late third instar stage for all GATC fragment mapped on dm6 genome annotation. The following values were attributed to the three states: enriched = 1, intermediate = 0, depleted = −1.

**Additional file 3: SF3.** WIG file containing the results of the three-state HMM analysis of *scrib*^*1*^ wing imaginal discs for all GATC fragment mapped on dm6 genome annotation. The following values were attributed to the three states: enriched = 1, intermediate = 0, depleted = −1.

**Additional file 4: Table S1.** Genes in Group I-IV; List of genes with number of GATC fragments within the presumptive regulatory region (2.5 kb upstream to 1.5 kb downstream of the transcriptional start sites (TSS)) displaying transitions between Pc-binding states (gain, loss or no-change transition between enriched, intermediate and depleted HMM states) in *scrib*^*1*^ if compared to WT profiles, and changes in gene expression levels of the respective gene in *scrib*^*1*^ to whose TSS the taGATC fragments had been mapped to. Group I (RNA – up regulated; Pc binding – gain); group II (RNA – down regulated; Pc binding – gain); group III (RNA – up regulated; Pc binding – loss); group IV (RNA – down regulated; Pc binding – loss); m1 (RNA – down regulated; Pc binding – loss and gain); m2 (RNA – up regulated; Pc binding – loss and gain).

**Additional file 5: Table S2** i-cisTarget Analysis of group II and III genes; List of regulatory elements identified by i-cisTarget that either represent transcription and chromatin-binding factors or specific histone modifications enriched within the presumptive regulatory region (2.5 kb upstream to 1.5 kb downstream of the transcriptional start sites (TSS)) of genes belonging to group II (RNA – down regulated; Pc binding – gain); group III (RNA – up regulated; Pc binding – loss).

**Additional file 6: SF4** WIG file containing the results of the three-state HMM analysis of wild type wing imaginal discs in early third instar stage for all GATC fragment mapped on dm6 genome annotation. The following values were attributed to the three states: enriched = 1, intermediate = 0, depleted = −1.

**Additional file 7: SF1** Script developed by A. Ivankin used to perform the three-state HMM analysis based on the previously published BioHMM algorithm (Marioni et al. [66]).

**Additional file 8.** Pc binding intensities (log2), normalized to Dam-binding and averaged between two replicates, and the corresponding results from the three-state HMM analysis for all three biological samples (early and lat wild type wing imaginal discs, and *scrib*^*1*^ wing imaginal discs) mapped on dm3 genome annotation.

**Additional file 9.** Not normalised read counts per GATC fragment for all sequenced samples mapped on dm3 genome annotation.
