# Correction to: DamID profiling of dynamic Polycomb-binding sites in *Drosophila* imaginal disc development and tumorigenesis

**DOI:** 10.1186/s13072-018-0206-0

**Published:** 2018-07-03

**Authors:** Marco La Fortezza, Giovanna Grigolon, Andrea Cosolo, Alexey Pindyurin, Laura Breimann, Helmut Blum, Bas van Steensel, Anne-Kathrin Classen

**Affiliations:** 1 Faculty of Biology, Ludwig-Maximilians-University Munich, Grosshaderner Strasse 2-4, 82152 Planegg, Martinsried, Germany; 2 Department of Environmental Systems Science, ETH Zurich, Universitätstrasse 16, 8092 Zurich, Switzerland; 3 Department of Health Sciences and Technology, ETH Zurich, Schorenstrasse 16, 8603 Schwerzenbach, Switzerland; 4 Center for Biological Systems Analysis, Albert-Ludwigs-University Freiburg, Habsburgerstrasse 49, 79104 Freiburg, Germany; 5 Institute of Molecular and Cellular Biology, Siberian Branch of Russian Academy of Sciences, Acad. Lavrentiev Ave. 8/2, Novosibirsk, 630090 Russia; 6 Max-Delbrück-Center for Molecular Medicine (MDC), Robert-Rössle-Str. 10, 13092 Berlin, Germany; 7 Laboratory for Functional Genome Analysis, Gene Center Munich, Ludwig-Maximilians-University Munich, Feodor-Lynen-Str. 25, 81377 Munich, Germany; 8 Division Gene Regulation, The Netherlands Cancer Institute, Plesmanlaan 121, 1066 CX Amsterdam, The Netherlands

## Correction to: Epigenetics & Chromatin (2018) 11:27 10.1186/s13072-018-0196-y

Unfortunately, the original version of this article contained a typographical error in one of the author names. The name of the author Alexey Pindyurin was incorrectly spelt as Alexey Pinduyrin. The correct spelling is included here and has been updated in the original article.
