# Supplementary material for: DamID profiling of dynamic Polycomb-binding sites in Drosophila imaginal disc development and tumorigenesis
Source: Epigenetics Chromatin. 2018 Jun 5;11:27. doi: 10.1186/s13072-018-0196-y (PMC5987561; doi:10.1186/s13072-018-0196-y)
Supplement: Supplementary file 1 — Additional file 1. Supplemental figures S1–S5. [file 13072_2018_196_MOESM1_ESM.pdf]

Supplementary Information

DamID-profiling of dynamic Polycomb-binding sites in *Drosophila* imaginal disc development and tumorigenesis

Marco La Fortezza, Giovanna Grigolon, Andrea Cosolo, Alexey Pinduyrin,
Laura Breimann, Helmut Blum, Bas van Steensel, Anne-Kathrin Classen

Supplementary File 1

SF1_HMM_3states_fit.model

Script developed by A. Ivankin used to perform the three-state HMM analysis based on the previously published BioHMM algorithm (Marioni et al, 2006).

Supplementary File 2, 3, 4

SF2_WT_late_Pc_DamID-seq_dm6.wig

SF3_scrib_Pc_DamID-seq_dm6.wig

SF4_ WT_early_Pc_DamID-seq_dm6.wig

WIG files containing the results of the three-states HMM analysis for all GATC fragment mapped on *dm6* genome annotation. The following values were attributed to the three states: *enriched* = 1, *intermediate* = 0, *depleted* = -1.

Supplementary Table 1

List of genes with number of GATC fragments within the presumptive regulatory region (2.5 kb upstream to 1.5 kb downstream of the transcriptional start sites (TSS)) displaying transitions between Pc-binding states (*gain*, *loss* or *no-change* transition between *enriched, intermediate* and *depleted* HMM states) in *scrib*^{1} if compared to WT profiles, and changes in gene expression levels of the respective gene in *scrib*^{1} to whose TSS the taGATC fragments had been mapped to. Group I (RNA – up regulated; Pc binding – gain); group II (RNA – down regulated; Pc binding – gain); group III (RNA – up regulated; Pc binding – loss); group IV (RNA – down regulated; Pc binding – loss); m1 (RNA – down regulated; Pc binding – loss and gain); m2 (RNA – up regulated; Pc binding – loss and gain).

Supplementary Table 2

List of regulatory elements identified by *i-cisTarget* that either represent transcription and chromatin-binding factors or specific histone modifications enriched within the presumptive regulatory region (2.5 kb upstream to 1.5 kb downstream of the transcriptional start sites (TSS)) of genes belonging to group II (RNA – down regulated; Pc binding – gain); group III (RNA – up regulated; Pc binding – loss)

Figure S1.

DamID profiling is suitable to study protein-DNA interaction in wing imaginal discs

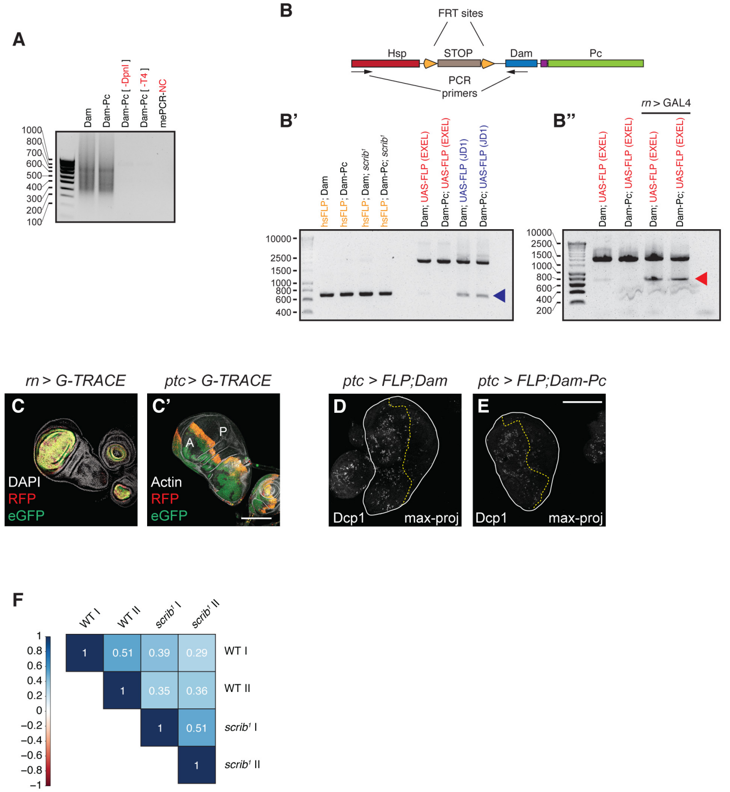

(A) Lanes 1 and 2: Characteristic DNA smear generated by the methylation-dependent PCR products if wing imaginal discs (WIDs) expressed Dam or Dam-Pc fusion proteins after induction of FLP expression by a heat-shock (*hsflp*). Negative controls in lanes 3-5 test the quality and specificity of the methylation-dependent PCR reaction.

(B) Schematic representation of the DamID construct and location of the standard PCR primers used to check for the genomic removal of the STOP-cassette after expression of a FLP.

(B') Lanes 1-4: Standard PCR products amplified from genomic DNA, if FLP was expressed ubiquitously in WIDs under the control of a heat shock-inducible *hsflp* (hsFLP). Only low molecular weight products (black arrowhead) indicating ubiquitous removal of the STOP-cassette are amplified. Lanes 5-8: Standard PCR products amplified from genomic DNA, if two different UAS-FLP constructs (FLP.JD1 and FLP.EXEL) were present in WIDs *in the absence of a GAL4 driver* and hence, should not be expressed. High molecular weight products are amplified for UAS-FLP.EXEL indicating that the STOP cassette is still intact. A proportion of low molecular weight products amplifying from WIDs carrying UAS-FLP.JD1 suggests that UAS-FLP.JD1 is expressed at low levels in the absence of GAL4 and cannot be used to achieve GAL4-dependent cell type specificity.

(B'') Standard PCR products amplified from genomic DNA, if UAS-FLP.EXEL was present in WIDs in the absence of a GAL4 driver (lanes 1 and 2) or in the presence of the *rotund* (*rn*)*GAL4* driver (lane 3 and 4). Partial removal of the STOP cassette reflects the restricted expression pattern of *rnGAL4* (see Fig. S1C).

(C-C') G-Trace labelling visualizes the historic (green) and real-time (red) expression of *rnGAL4* (C) and *patched* (*ptc*)*GAL4* (C') cell lineages in WIDs. A and P refer to anterior and posterior compartments, respectively.

(D-E) Staining for activated Dcp-1 in wing imaginal discs expressing FLP;Dam (D) or FLP;Pc-Dam (E) under control of *ptcGAL4*. To visualize expression patterns of the Myc-tagged Dam and DamPc proteins (see Fig.1C,D), abnormally high expression levels were induced by a 1 h heat-shock 6 h before dissection. Yellow dashed line indicates the anterior (left) / posterior (right) boundary. Maximum projections of a confocal stack are shown to reveal all signals.

(F) Pearson's correlations between two biological replicates of DamID-seq Pc-binding profiles obtained for WT and *scrib*^{1} WIDs.

All scale bars: 100 µm.

Figure S2.

DamID-seq workflow

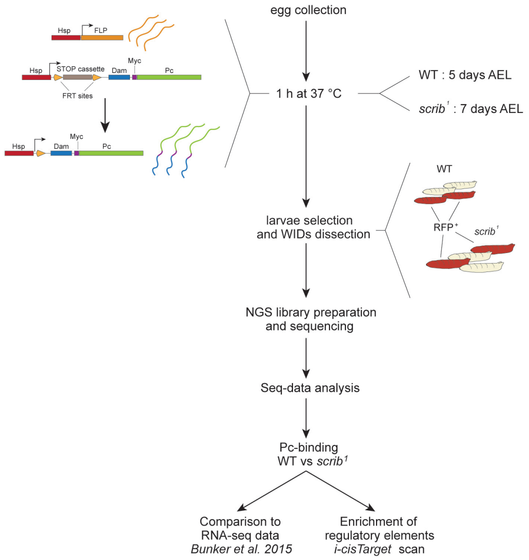

Representation of the workflow applied to characterize differences in Pc-binding sites between *scrib*[1] and WT WIDs using DamID-seq. Transgenic larvae carrying the Dam or DamPc proteins can be distinguished from other genotypes by the presence of the 3xP3-RFP reporter included in the transgenic landing sites (ZH-51C) used for the genomic integration of DamID vectors.

Figure S3.

Reproducibility of PCR-free NGS DamID-Seq libraries

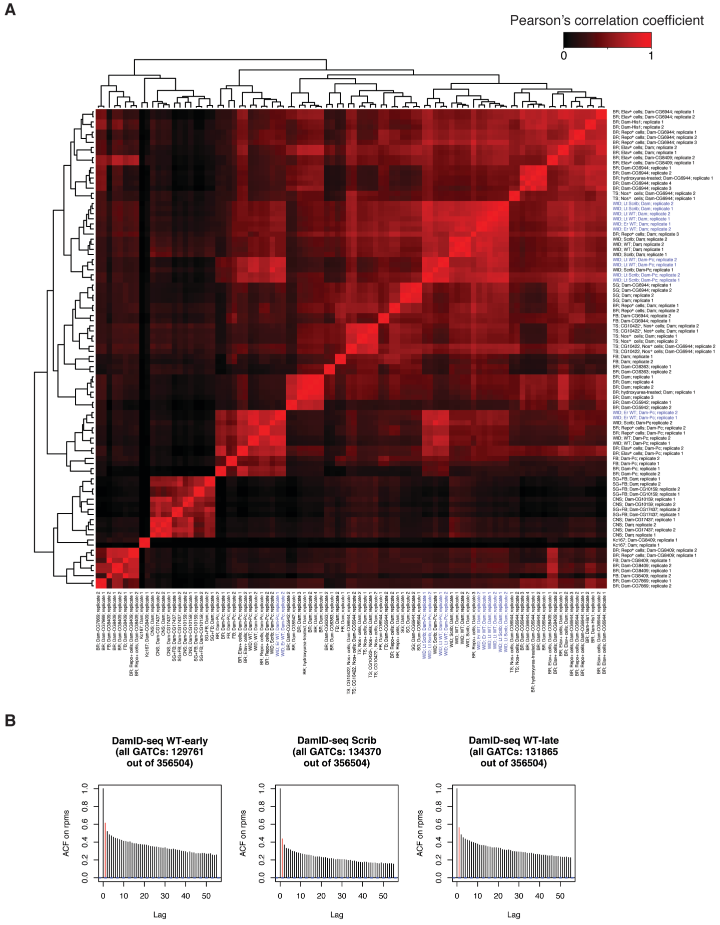

(A) Hierarchical clustering heatmap of Pearson's correlation coefficients between 94 DamID-seq samples generated for a number of Drosophila proteins and cell types (unpublished). The samples are named as follows: tissue or cell culture; cell type within

the tissue and/or mutant background (optional); Dam fusion protein; DamID replicate number. BR, third instar larval central brain; CNS, third instar larval central nervous system; FB, third instar larval fat bodies; SG, third instar larval salivary glands; TS; adult testes; WID, wing imaginal discs. Note that the replicates of each WID DamID-seq experiment generated and used in this study (highlighted in blue) cluster together.

(B) Autocorrelation analysis of Pc-binding intensities at neighboring GATC fragments performed on normalized and averaged biological replicates for 3 DamID-Seq data sets (early or late WT WID, *scrib^1* WID) used in this study. Note that the autocorrelation of neighboring GATC sites at Lag 2 (red vertical line in the graphs) should be higher than 0.3 to consider the biological replicates reproducible [1].

1. van Bemmel JG, Filion GJ, Rosado A, Talhout W, de Haas M, van Welsem T, van Leeuwen F, van Steensel B: **A network model of the molecular organization of chromatin in Drosophila**. *Mol Cell* 2013, **49**(4):759-771.

Figure S4.

Polycomb-binding is altered at specific *loci* in *scrib^1* wing discs

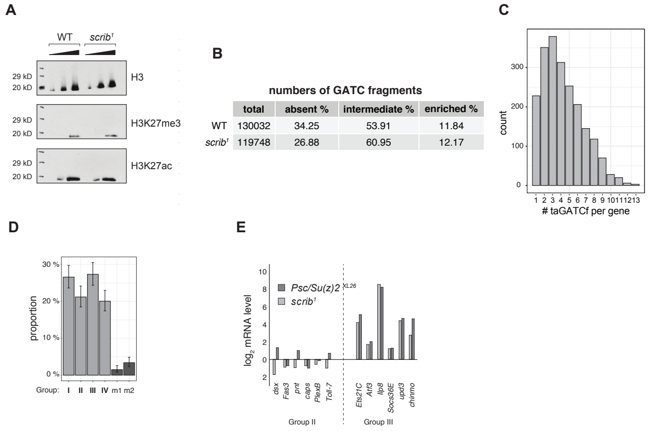

(A) Western blot analysis of H3, H3K27me3 and H3K27ac histone modification levels in WT and *scrib^1* WIDs. Black triangle indicates the increase in sample loaded per lane.

(B) Number of GATC fragments per genome analyzed in DamID-Seq samples from WT and *scrib^1* WIDs and the distribution of their classification after a three-state HMM analysis.

(C) Bar plot representing the distribution of the counts of genes differentially expressed in *scrib^1* compared to WT as a function of the number of transcription-associated GATC fragments (taGATCf) that mapped to the regulatory region of these genes.

(D) Bar graph representing the proportion of taGATCf mapped genes differentially expressed in *scrib^1* classified into group I, II, III, or IV. Only a minor proportion of genes whose expression is decreased (m1) or increased (m2) in *scrib^1* shows both *gain* and *loss* transitions for taGATCf at the same presumptive regulatory region surrounding the TSS.

(E) Bar plot of changes in transcription levels of indicated genes in group II or III in imaginal discs mutant for *Psc-Su(z)2^{XL26}* (dark grey) or *scrib^1* (light grey) obtained from (Bunker et al. 2015).

Figure S5.

GATC-fragments size depends on developmental stage in DamID-seq Pc-profiles

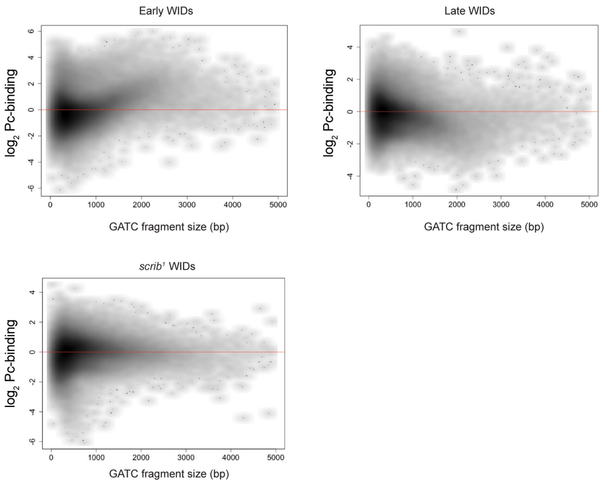

Scatter plots displaying the $\log_2$ of Pc-binding intensity as function of the GATC-fragment size for WIDs in early development, WIDs in late development and *scrib*^{1} WIDs. Note the shape of the clouds in all three different samples.
